# The impact of the interaction between sarcopenia and frailty on mortality in older adult with cardiovascular disease

**DOI:** 10.1093/geroni/igaf122.2895

**Published:** 2025-12-31

**Authors:** Alberto Frisoli, Amanda Diniz, Giovana Menin

**Affiliations:** Universidade Federal de Sao Paulo, São Paulo, Sao Paulo, Brazil; Universidade Federal de Sao Paulo, São Paulo, Sao Paulo, Brazil; Universidade Federal de Sao Paulo, São Paulo, Sao Paulo, Brazil

## Abstract

**Introduction:**

Sarcopenia and physical frailty are very prevalent in the elderly with cardiovascular disease (CVD) and increase the risk of mortality. However, it is still unclear whether there is an interaction between them in relation to mortality or whether they act independently.

**Objective:**

To assess the predictive value of sarcopenia and frailty in the mortality of older adults with CVD.

**Methods:**

Longitudinal analysis of SARCOS, an epidemiological study on sarcopenia and osteoporosis for mortality in the older adults with CVD. Ethic committee - CAE:24412713.6.0000.5505. Sarcopenia was diagnosed by the SDOC recommendation and frailty by the Hopkins criteria at the beginning of the study. Mortality was assessed by telephone call at 6-12-18 months. Survival analyses were performed by Cox regression (p < 0.08).

**Results:**

In 496 subjects, the mean age was 77.85 (±7.8) and 56.7% were women (p.ns). The mortality rate was 8.1% (n = 40), and among those who died, 67.5% (n = 27) had frailty, 27.5% (n = 11) pre-frailty (p < 0.001) and 65% (n = 26) sarcopenia (p = 0.001). Heart failure, atrial fibrillation, CRF, age, stroke, hospitalizations, falls, and alcohol consumption were also associated with mortality (p < 0.05). The interaction between sarcopenia and frailty was significant (p < 0.001). In the adjusted multivariate analysis for the variables and the interaction between sarcopenia and frailty, frailty showed HR: 4.16 (1.12-15.42; p = 0.033) and sarcopenia HR: 4.13 (0.95-17.95; p = 0.058). In the subtraction model of the non-significant variables frailty remained significant with HR 3.80 (1.11-13.01; p = 0.033).

**Conclusions:**

In older adults with CVDs, frailty and sarcopenia had similar mortality risk values, regardless of the interaction between them

